# Audiometric indicators of cochlear involvement in chronic suppurative otitis media with and without cholesteatoma

**DOI:** 10.1007/s00405-026-10465-9

**Published:** 2026-07-27

**Authors:** Ahmed Mohammed Abdelghany, Osama Ahmed Ali Elsayad, Fatma Gamal Abdelmonged Saleh, Noha Mohamed Magdi Zaki

**Affiliations:** 1 Otorhinolaryngology Department, Benha University, Farid Nada Street, Benha, Qalyubia 13518 Egypt; 2 Faculty of Medicine Benha University, Benha, Egypt

**Keywords:** Chronic Suppurative Otitis Media, Cholesteatoma, Mixed Hearing Loss

## Abstract

**Purpose:**

This study aimed to compare cochlear function in patients with chronic suppurative otitis media (CSOM) with and without cholesteatoma and to identify clinical predictors of mixed hearing loss.

**Methods:**

A retrospective comparative study conducted at the Otorhinolaryngology Department, Benha University Hospitals, Egypt, from April 2024 to April 2025. Sixty-two patients with unilateral CSOM and complete audiologic and radiologic data were included, comprising 30 with cholesteatoma and 32 without. Evaluations included pure-tone audiometry, speech reception threshold (SRT), speech discrimination score (SDS), and temporal bone computed tomography. Cholesteatoma severity was staged using the EAONO/JOS classification. Multivariate regression analyses were performed to identify factors associated with mixed hearing loss and air- and bone-conduction pure-tone averages (PTA), with bone-conduction PTA serving as a surrogate for cochlear involvement.

**Results:**

Disease duration was significantly longer in the cholesteatoma group (*P* = 0.023), while other baseline characteristics were comparable. Hearing metrics (PTA, SRT, SDS) did not differ significantly between groups. Multivariate analysis identified advancing age and longer disease duration as independent predictors of mixed hearing loss, whereas the presence of cholesteatoma was not.

**Conclusion:**

Cholesteatoma was associated with longer disease duration but was not independently associated with measurable cochlear involvement, based on the audiometric measures and cross-sectional design employed. Age and disease chronicity were the primary determinants of audiometric patterns compatible with cochlear involvement, underscoring the importance of early diagnosis and timely management.

## Introduction

Otitis media is among the most common causes of hearing impairment across all age groups. Chronic suppurative otitis media (CSOM) represents a persistent stage of middle-ear inflammation characterized by long-standing otorrhea through a non-intact tympanic membrane [1]. Beyond its burden of recurrent infection, CSOM is associated with a variety of sequelae—including aural polyps, osteitis, tympanosclerosis, labyrinthitis, and potentially serious intracranial complications—that collectively contribute to substantial morbidity [2]. Although the resulting hearing loss is typically conductive due to tympanic membrane perforation and ossicular pathology, prolonged inflammation may also affect the inner ear. Repeated infectious episodes allow inflammatory mediators, bacterial toxins, and other macromolecules to diffuse across the round window membrane, potentially damaging cochlear hair cells and producing sensorineural hearing loss (SNHL) in a subset of patients [3].

Cholesteatoma represents a more aggressive form of chronic middle-ear disease. As a keratinizing epithelial mass, it progressively expands within the tympanomastoid compartment and frequently harbors persistent bacterial infection. Its invasive behavior and propensity for bony erosion commonly lead to conductive hearing loss through ossicular chain destruction [4]. Given its locally destructive nature, cholesteatoma has long been suspected to exacerbate inner-ear involvement [5].

However, whether cholesteatoma confers additional cochlear involvement beyond that associated with non-cholesteatomatous CSOM remains unresolved. Published studies have yielded conflicting results: some report higher rates of SNHL in the presence of cholesteatoma [6, 7], while others find no significant differences in bone-conduction thresholds between cholesteatomatous and non-cholesteatomatous ears [8, 9]. This inconsistency highlights the need for further investigation using physiologically grounded audiometric approaches.

Accordingly, this study aimed to compare cochlear involvement in patients with CSOM with and without cholesteatoma using objective audiometric surrogates of inner-ear function. In addition, we sought to determine whether cholesteatoma independently predicts mixed hearing loss and to identify clinical factors associated with cochlear impairment in chronic ear disease.

## Patients and methods

### Study design and setting

This retrospective cross-sectional comparative study was conducted at the Otorhinolaryngology Department, Benha University Hospitals, Egypt, between April 2024 and April 2025, using single-time-point clinical, audiometric, and radiologic assessments of patients with chronic suppurative otitis media with and without cholesteatoma. Given the retrospective cross-sectional design, findings represent single-time-point audiometric associations and do not permit conclusions regarding progression or causality.

### Study population

Patients aged 10–60 years diagnosed with CSOM were included. CSOM was defined as middle-ear inflammation and/or otorrhea persisting for at least 12 weeks, the presence of a non-intact tympanic membrane on otoscopic or microscopic examination, and supportive findings on high-resolution temporal bone computed tomography (CT) [10]. Disease duration was obtained from documented medical records when available and supplemented by patient or caregiver report at the time of evaluation. As a retrospective measure, duration represents an approximate estimate and may be subject to recall bias.

### Inclusion criteria

Inclusion required the availability of complete audiologic data, including pure-tone audiometry, speech reception threshold (SRT), and speech discrimination score (SDS), as well as high-resolution temporal bone computed tomography (CT). *Eligible participants had unilateral chronic suppurative otitis media with conductive or mixed hearing loss*. All included participants were able to undergo standard pure-tone and speech audiometry, and no preschool-aged children were enrolled.

### Exclusion criteria

Included pure sensorineural hearing loss, bilateral chronic ear disease, a history of ototoxic drug exposure, significant noise exposure or acoustic trauma, systemic or metabolic disorders associated with ototoxicity, previous meningitis or childhood exanthematous diseases affecting hearing, a family history of congenital or early-onset sensorineural hearing loss, significant head trauma, prior ear surgery, active acute otitis media at the time of testing, or incomplete medical, audiologic, or radiologic records. Bilateral cases were excluded to avoid confounding of ear-specific audiometric outcomes and to allow accurate attribution of cochlear findings to the affected ear.

### Group classification and disease assessment

Patients were classified into two groups: CSOM with cholesteatoma and CSOM without cholesteatoma. *Cholesteatoma* was diagnosed based on otomicroscopic examination and characteristic CT findings and was staged according to the EAONO/JOS classification [4, 5]. *Hearing loss type* was classified in accordance with established criteria [11]. *Conductive hearing loss* (CHL) was defined as bone-conduction (BC) thresholds ≤ 25 dB HL across tested frequencies with an air–bone gap (ABG) ≥ 10 dB at two or more frequencies. *Mixed hearing loss* was defined as BC thresholds > 25 dB HL at two or more frequencies in the presence of an ABG ≥ 10 dB at two or more frequencies.

*The pure-tone average (PTA)* for the tested ear was calculated as the arithmetic mean of air-conduction thresholds at 0.5, 1, 2, and 4 kHz (AC-PTA). Additionally, a bone-conduction PTA (BC-PTA) was calculated as the arithmetic mean of BC thresholds at 0.5, 1, 2, and 4 kHz. AC-PTA was used to quantify functional hearing impairment, representing the cumulative effects of middle-ear and cochlear pathology. In contrast, BC-PTA was analyzed as a complementary outcome to provide an audiometric surrogate of cochlear involvement by reducing the influence of conductive deficits.

*The air–bone gap (ABG)* was calculated as the difference between air-conduction and bone-conduction pure-tone averages (AC-PTA − BC-PTA) at 0.5, 1, 2, and 4 kHz for the affected ear.

*Hearing loss degree* was then categorized based on the affected ear AC-PTA as follows: slight (16–25 dB HL), mild (26–40 dB HL), moderate (41–55 dB HL), moderately severe (56–70 dB HL), severe (71–90 dB HL), and profound (≥ 91 dB HL) [12].

*Audiologic data* were extracted from medical records, with documentation confirming standardized testing using calibrated equipment and appropriate masking. Bone-conduction thresholds were obtained using standard clinical bone vibrators with appropriate contralateral masking applied whenever indicated. Because BC thresholds in CSOM may be influenced by middle-ear mechanics (Carhart effect), a sensitivity analysis was performed in which a 10 dB correction at 2 kHz was applied in cases with suspected ossicular discontinuity, defined by large air–bone gaps and/or radiologic evidence on CT.

*Speech audiometry* was performed using recorded standardized Arabic materials under calibrated conditions, with appropriate contralateral masking applied according to standard clinical criteria when indicated. All participants completed testing reliably; however, as in routine clinical practice, speech measures in younger patients may exhibit greater variability.

*High-resolution temporal bone CT* with submillimetric sections was obtained as part of standard clinical evaluation.

### Statistical analysis

Statistical analysis was performed using SPSS version 27 (IBM Corp., Armonk, NY, USA). Continuous variables were summarized as means ± standard deviations or medians with ranges, while categorical variables were presented as frequencies and percentages. Group comparisons were conducted using independent t-tests or Mann–Whitney U tests for continuous variables and Chi-square or Fisher’s exact tests for categorical variables. Correlations were assessed using Spearman’s correlation coefficient. Correlation analysis was used to characterize associations between audiometric measures and clinical variables, providing complementary analytical context for subsequent multivariate modeling. Multivariate logistic regression was used to identify predictors of mixed hearing loss, and multivariate linear regression was used to identify predictors of PTA. A two-sided P value < 0.05 was considered statistically significant. Given the relatively modest sample size, the robustness of the logistic regression model is limited. The events-per-variable ratio was approximately 4, which is below the conventional threshold of 10. These findings should therefore be interpreted with caution, and replication in larger cohorts is warranted.

## Results

### Study population and baseline characteristics

This study included 62 patients with chronic suppurative otitis media (CSOM), comprising 30 with cholesteatoma and 32 without. There were no significant differences between groups with respect to age (median: 30 vs. 38 years; *P* = 0.607), sex distribution (*P* = 0.622), or affected ear side (*P* = 0.321). Patients with cholesteatoma had a significantly longer duration of ear disease than those without cholesteatoma (median: 5 vs. 3 years; *P* = 0.023). Among cholesteatoma cases, severity was stage I in 40.0%, stage II in 53.3%, and stage III in 6.7% (Table 1).

**Table 1:** Baseline and clinical characteristics of patients with and without cholesteatoma

Variable	CSOM with cholesteatoma (*n* = 30)	CSOM without cholesteatoma (*n* = 32)	*P* value
Age (years), median (range)	30 (10–56)	38 (12–60)	0.607
Gender, n (%)			
Male	15/30 (50.0%)	18/32 (56.3%)	0.622
Female	15/30 (50.0%)	14/32 (43.8%)	
Affected ear side, n (%)			
Right	15/30 (50.0%)	20/32 (62.5%)	0.321
Left	15/30 (50.0%)	12/32 (37.5%)	
Duration of ear disease (years), median (range)	5 (1–30)	3 (1–40)	0.023**
*Cholesteatoma severity, n (%)			
Stage I	12/30 (40.0%)	—	
Stage II	16/30 (53.3%)	—	
Stage III	2/30 (6.7%)	—	

* Percentages and severity calculated among patients with cholesteatoma only

** *P* < 0.05

### Hearing loss characteristics according to cholesteatoma presence

The distribution of hearing loss type did not differ significantly between groups (*P* = 0.48), with conductive hearing loss predominating in both cohorts. Pure-tone average values were comparable between patients with and without cholesteatoma (*P* = 0.83 for AC-PTA and *P* = 0.72 for BC-PTA), and no significant difference was observed in hearing loss severity distribution (*P* = 0.35).

The mean air–bone gap (ABG) was significantly higher in the cholesteatoma group compared with the non-cholesteatomatous group (29 ± 7 dB vs. 22 ± 6 dB, *P* < 0.001) (Table 2).

**Table 2:** Hearing loss characteristics according to cholesteatoma presence

Variable	CSOM with cholesteatoma (*n* = 30)	CSOM without cholesteatoma (*n* = 32)	*P* value
Type of hearing loss, *n* (%)			
Conductive HL	19/30 (63.3%)	23/32 (71.9%)	0.48
Mixed HL	11/30 (36.7%)	9/32 (28.1%)	
Pure Tone Average (PTA)			
* AC-PTA (dB HL), median (range)	45 (25–90)	45 (20–100)	0.83
** BC-PTA (dB HL), median (range)	35 (20–60)	26 (15–55)	0.22
ABG (dB), mean ± SD	29 ± 7	22 ± 6	< 0.001 ***
Degree of hearing loss ****, n (%)			
Slight	1/30 (3.3%)	3/32 (9.4%)	0.35
Mild	12/30 (40.0%)	11/32 (34.4%)	
Moderate	12/30 (40.0%)	7/32 (21.9%)	
Moderately severe	4/30 (13.3%)	6/32 (18.8%)	
Severe	1/30 (3.3%)	2/32 (6.3%)	
Profound	0/30 (0.0%)	3/32 (9.4%)	

* AC-PTA = Air-conduction Pure Tone Average

** BC-PTA = Bone-conduction Pure Tone Average

*** *P* < 0.001

**** Degree categorized based on AC-PTA

### Speech audiometry findings

Speech audiometric measures were similar between groups. No statistically significant differences were observed in speech reception threshold (SRT, *P* = 0.67) or speech discrimination score (SDS, *P* = 0.13) between patients with and without cholesteatoma (Table 3).

**Table 3:** Speech audiometry findings in patients with and without cholesteatoma

Parameter	CSOM with cholesteatoma (*n* = 30)	CSOM without cholesteatoma (*n* = 32)	*P* value
Speech reception threshold (dB), median (range)	35 (25–75)	40 (20–95)	0.67
Speech discrimination score (%), mean ± SD	93 ± 10	88 ± 16	0.13

### Correlation analysis

Correlation analysis demonstrated significant positive associations between AC-PTA and age (*r* = 0.513, *P* < 0.001), disease duration (*r* = 0.291, *P* = 0.02), SRT (*r* = 0.845, *P* < 0.001), and cholesteatoma severity (*r* = 0.511, *P* < 0.001). AC-PTA was negatively correlated with SDS (*r* = − 0.651, *P* < 0.001) (Table 4).

**Table 4:** Correlation between pure tone averages (AC, BC) and clinical/audiological parameters

Parameter	AC-PTA		BC-PTA	
	*r*	*P* value	*r*	*P* value
Age (years)	0.513	< 0.001*	0.430	< 0.001*
Duration of ear disease (years)	0.291	0.022*	0.621	0.002*
Speech reception threshold (dB)	0.845	< 0.001*	0.435	< 0.001*
Speech discrimination score (%)	−0.651	< 0.001*	−0.881	< 0.001*
Cholesteatoma severity	0.511	< 0.001*	0.661	< 0.001*

*P < 0.05

**Fig. 1:**
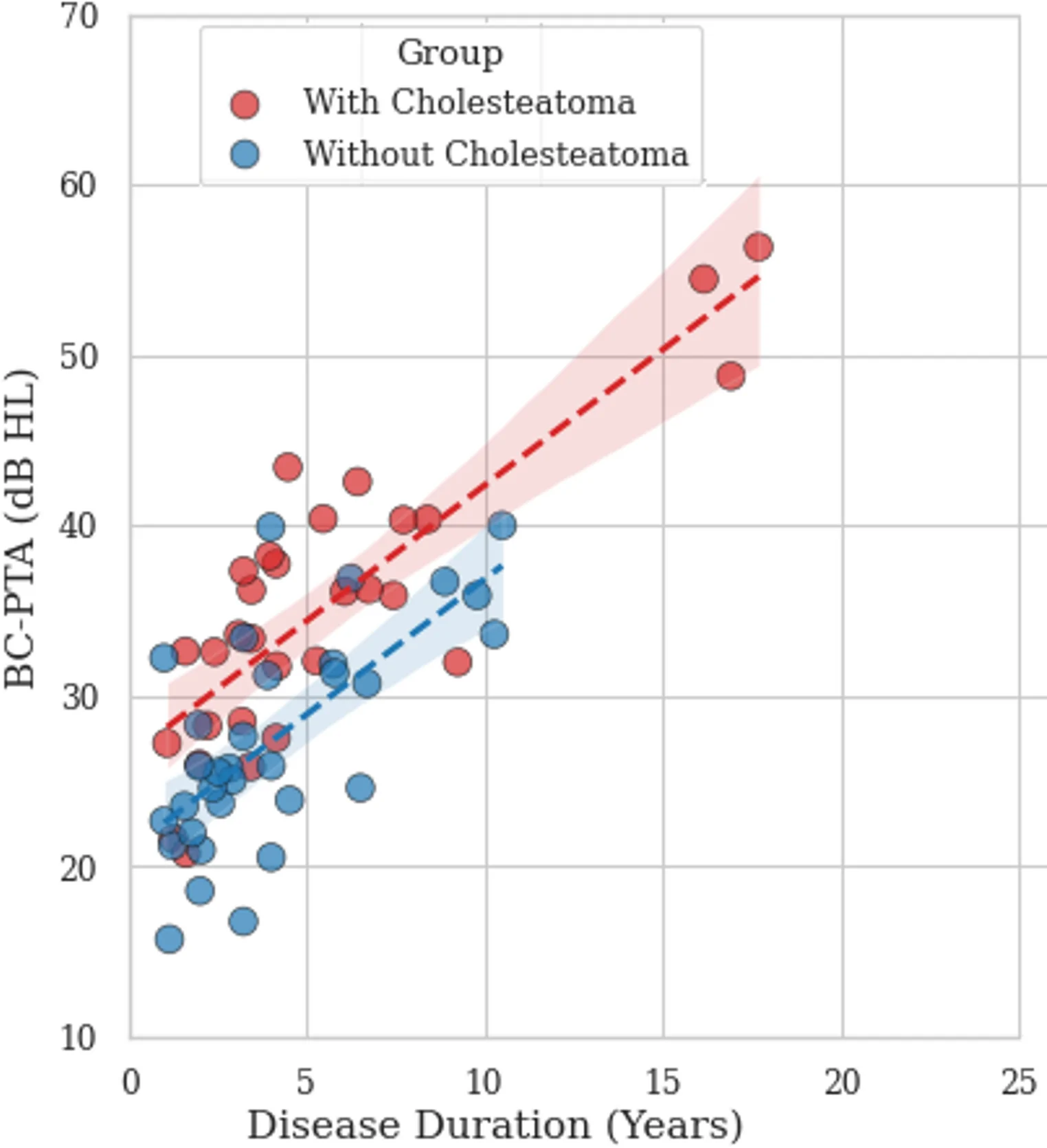
Relationship between disease duration and bone-conduction pure-tone average (BC-PTA). Scatter plot illustrating the association between disease duration (years) and BC-PTA (dB HL), stratified by cholesteatoma presence (red = with cholesteatoma; blue = without cholesteatoma). Dashed lines represent group-specific linear regression trends. Overall correlation: *r* = 0.621, *P* = 0.002

Using BC-PTA as an audiometric surrogate of cochlear involvement, strong correlations were observed with disease duration (*r* = 0.621, *P* = 0.002) (Fig. 1) and cholesteatoma severity (*r* = 0.661, *P* < 0.001). BC-PTA also demonstrated a moderate positive correlation with SRT (*r* = 0.435, *P* < 0.001) and a strong inverse correlation with SDS (*r* = − 0.881, *P* < 0.001), while age showed a weaker but statistically significant association (*r* = 0.430, *P* < 0.001) (Table 4).

Although cholesteatoma severity demonstrated significant correlations with cochlear-weighted audiometric measures, it was not included as an independent variable in multivariate regression models.

### Multivariate regression analysis

Multivariate logistic regression identified increasing age (OR = 1.103, *P* = 0.006) and longer disease duration (OR = 1.246, *P* = 0.026) as independent predictors of mixed hearing loss, whereas cholesteatoma presence was not independently associated with this outcome (*P* = 0.09).

In multivariate linear regression with AC-PTA as the dependent variable, longer disease duration independently predicted higher AC-PTA values (B = 0.419 dB HL per year, *P* = 0.019). Cholesteatoma presence was not an independent predictor.

When BC-PTA was used as the dependent variable to better reflect cochlear-weighted involvement, cholesteatoma presence likewise did not independently predict BC-PTA, whereas longer disease duration remained a significant determinant. Application of a 2-kHz Carhart correction in cases with suspected ossicular discontinuity did not materially alter these results (Table 5).

**Table 5:** Multivariate regression analyses of factors associated with cochlear involvement

Variable	Mixed hearing loss (Logistic regression) OR (95% CI)	*P* value	PTA (Linear regression)			
			AC-PTA B (95% CI)	*P* value	BC-PTA B (95% CI)	*P* value
Age (years)	1.10 (1.03–1.18)	0.006*	0.18 (− 0.00 to 0.36)	0.053	0.19 (− 0.02 to 0.56)	0.074
Gender	1.35 (0.76–3.22)	0.237	2.02 (–3.22 to 4.27)	0.71	1.72 (–2.82 to 5.07)	0.503
Affected ear side	1.19 (0.92–1.53)	0.118	1.14 (–0.48 to 2.21)	0.26	1.84 (–0.68 to 2.89)	0.762
Cholesteatoma presence	1.10 (0.95–1.44)	0.09	1.58 (–1.50 to 2.06)	0.76	1.15 (–1.23 to 1.36)	0.528
Duration of ear disease (years)	1.25 (1.03–1.52)	0.026*	0.42 (0.09 to 0.52)	0.019*	0.65 (0.36 to 1.16)	0.005*

*OR* odds ratio, *B* regression coefficient, *CI* confidence interval, *AC-PTA* air-conduction pure-tone average (functional hearing), *BC-PTA* bone-conduction pure-tone average (audiometric surrogate of cochlear involvement). *P < 0.05

Logistic regression outcome: mixed hearing loss (yes/no)

Linear regression outcome: pure tone average

## Discussion

Chronic suppurative otitis media (CSOM) remains a major contributor to preventable hearing impairment worldwide. Prolonged inflammatory activity may extend beyond the middle ear and involve cochlear structures [1, 13]. Whether cholesteatoma, as a more aggressive manifestation of chronic middle-ear disease, confers additional risk of cochlear impairment beyond non-cholesteatomatous CSOM remains controversial [5, 7–9]. The present study addressed this issue by comparing audiologic outcomes between patients with and without cholesteatoma and by identifying clinical predictors of mixed hearing loss and audiometric indicators of cochlear involvement. Given the cross-sectional design, these findings should be interpreted as associative rather than causal.

Cholesteatoma was associated with a significantly longer duration of ear disease, suggesting that longstanding middle-ear pathology may predispose to cholesteatoma formation rather than implying that cholesteatoma itself prolongs disease. This observation is consistent with prior descriptions of cholesteatoma as the culmination of chronic inflammatory and epithelial remodeling processes within the middle ear [5]. Similar relationships between disease chronicity and structural deterioration have been reported by Khan et al. [7] and Islam et al. [14], reinforcing the concept that longer-standing disease reflects cumulative middle-ear pathology.

Despite the longer disease duration, cholesteatoma presence was not associated with worse pure-tone thresholds, poorer speech outcomes, or a higher prevalence of mixed hearing loss in our cohort. These findings align with reports by Amali et al. [8] and Sharma and Sharma [9], who likewise observed no significant differences in bone-conduction thresholds between cholesteatomatous and non-cholesteatomatous CSOM. Multivariate analysis further demonstrated that cholesteatoma did not independently predict mixed hearing loss after adjustment for age and disease duration. The relatively narrow confidence interval (0.95–1.44) suggests that any independent effect, if present, is likely modest rather than clinically substantial. Together, these findings indicate that audiometric patterns compatible with cochlear involvement are more closely associated with chronic disease exposure than with cholesteatoma presence alone.

Several studies have reported higher rates of mixed hearing loss in cholesteatomatous disease [6, 7, 15], highlighting ongoing debate. Differences in study design, population characteristics, and analytic approaches likely contribute to these discrepancies.

A central finding of this study is the strong association between audiometric surrogates of cochlear involvement and both advancing age and longer disease duration. Age emerged as the strongest predictor of mixed hearing loss, while disease duration independently predicted higher pure-tone averages. These observations are consistent with findings by Papp et al. [15] and Redaelli de Zinis et al. [16], who reported associations between prolonged middle-ear inflammation and deterioration of cochlear thresholds. Experimental and clinical data support the biological plausibility that repeated exposure to inflammatory mediators crossing the round window membrane may contribute to cumulative inner-ear changes over time [13, 17].

From a clinical perspective, distinguishing overall hearing impairment from cochlear-weighted involvement has practical implications. Air-conduction PTA reflects functional hearing disability, whereas bone-conduction PTA more closely approximates inner-ear contribution and may assist in prognostic counseling regarding postoperative or rehabilitative outcomes. Although the cholesteatoma group demonstrated a significantly larger air–bone gap—consistent with greater conductive pathology—bone-conduction thresholds were not independently worse. This pattern indicates that cochlear-weighted audiometric changes were more strongly related to disease chronicity than to cholesteatoma presence alone. Importantly, BC-PTA does not directly diagnose cochlear pathology and may remain influenced by middle-ear mechanics despite corrective adjustments; therefore, these findings should be interpreted as functional audiometric indicators rather than definitive evidence of histopathologic injury. Accordingly, the conclusions of this study are best framed as reflecting a lack of independent association between cholesteatoma and cochlear-weighted audiometric measures, rather than a definitive absence of cochlear effect, given the inherent limitations of BC-PTA as an indirect surrogate for cochlear function.

Speech audiometry provided complementary functional insight beyond tonal thresholds. While pure-tone measures quantify auditory sensitivity, speech discrimination more directly reflects cochlear and neural integrity. In the present study, BC-PTA showed a strong inverse correlation with speech discrimination score, indicating that worsening cochlear-weighted thresholds were associated with reduced speech perception. This relationship supports the interpretation that inner-ear involvement, rather than conductive dysfunction alone, contributes to impaired auditory clarity in chronic disease.

The absence of an independent association between cholesteatoma and speech audiometric measures further supports the view that cochlear-related functional impairment in CSOM is more closely linked to disease chronicity than to cholesteatoma itself. Similar observations were reported by Popescu et al. [18], who found poorer auditory performance in patients with mixed hearing loss compared with those with purely conductive loss. This pattern is biologically plausible, particularly in older individuals with reduced cochlear reserve, in whom prolonged inflammatory exposure may exert greater functional impact.

Although our findings are consistent with several previous studies, the literature remains heterogeneous. Some reports have not demonstrated significant associations between age, disease duration, and sensorineural hearing loss, possibly reflecting differences in sample size, population characteristics, or analytic strategies, including reliance on contralateral-ear controls in cohorts with prevalent bilateral disease [6, 7, 15]. Nevertheless, the convergence of tonal, speech, and multivariate analyses in the present study strengthens the interpretation that aging and disease chronicity are closely associated with audiometric patterns compatible with cochlear involvement.

Beyond regression modeling, correlation analysis provided complementary insight into functional relationships between audiometric measures and clinical variables. The associations between BC-PTA, disease duration, and speech performance reinforce the internal coherence of these findings, while the divergence between correlation and multivariate results for cholesteatoma severity highlights the distinction between association and independent prediction.

Parallel findings across AC-PTA and BC-PTA models indicate that the lack of an independent association with cholesteatoma was observed in both overall hearing performance and cochlear-weighted thresholds after adjustment for disease duration. Cholesteatoma severity was not included in regression analyses because staging applies exclusively to cholesteatomatous ears and cannot be uniformly modeled across the cohort. Inclusion of severity would have limited analysis to a subgroup and introduced collinearity with disease duration, thereby reducing interpretability. Accordingly, severity was evaluated descriptively and through correlation analyses rather than entered as an independent predictor. Importantly, the strong correlation observed between cholesteatoma severity and BC-PTA (*r* = 0.661, *P* < 0.001) warrants further comment. Within the cholesteatoma subgroup, higher stage disease was associated with greater cochlear-weighted audiometric deterioration. This relationship most plausibly reflects the confounding influence of disease duration: more advanced-stage cholesteatoma tends to represent longer-standing, progressive disease, which itself is the primary driver of cumulative cochlear-weighted change identified in multivariate analysis. This interpretation is consistent with the concept of cholesteatoma as the end-stage of chronic inflammatory and epithelial remodeling processes, rather than an independent cause of cochlear injury per se.

### Strengths and limitations

This study has several strengths. Cochlear-weighted involvement was assessed using both tonal and speech audiometric measures, allowing comprehensive evaluation of auditory performance and differentiation between functional impairment and inner-ear contribution. While air-conduction thresholds reflect patient-perceived disability, bone-conduction pure-tone averages provided an audiometric surrogate of cochlear involvement, reducing the likelihood of attributing conductive pathology to inner-ear dysfunction. Speech audiometry—particularly speech discrimination—offered additional insight into auditory clarity and neural processing. Cholesteatoma diagnosis and staging were based on otomicroscopy and high-resolution computed tomography using the EAONO/JOS classification, ensuring objective disease characterization. Multivariate analyses were performed to adjust for key confounders, enabling differentiation between the relative contributions of cholesteatoma presence, disease chronicity, and age. Several limitations should be acknowledged. The retrospective cross-sectional design permits evaluation of single-time-point associations but does not allow inference regarding progression or causality. Disease duration was partially based on patient recall, introducing potential recall bias and imprecision in estimating cumulative inflammatory exposure. The broad age range (10–60 years) introduces physiologic variability, and nonlinear age-related effects cannot be excluded despite statistical adjustment. Although cholesteatoma severity was staged using the EAONO/JOS classification and demonstrated significant correlations with audiometric measures, further stratification according to specific destructive features—such as ossicular erosion or labyrinthine involvement—was not available and may influence cochlear-weighted outcomes. In addition, this was a single tertiary-center study, which may limit generalizability to populations with different demographic or healthcare characteristics. Audiologic assessment was limited to a single time point, and longitudinal follow-up data were not available to evaluate potential progression of cochlear-weighted impairment. Finally, bone-conduction thresholds in CSOM may be influenced by middle-ear mechanics, potentially elevating BC values independent of true cochlear pathology and obscuring subtle deficits. To address this, a sensitivity analysis applying a 10 dB correction at 2 kHz was performed in cases with suspected mechanical artifact based on audiometric and radiologic criteria. Persistence of findings after this adjustment increases confidence in the observed associations; however, BC-based measures should be interpreted as clinical surrogates rather than definitive evidence of histopathologic injury, and mild cochlear dysfunction may remain underestimated or misclassified [16, 17].

## Conclusion

In patients with chronic suppurative otitis media, cholesteatoma presence was not independently associated with worse functional hearing, mixed hearing loss, or audiometric patterns compatible with cochlear involvement, based on the audiometric measures and cross-sectional design employed. These findings should be interpreted as reflecting a lack of independent association rather than a definitive absence of cochlear effect, given the inherent limitations of audiometric surrogates. Advancing age and longer disease duration were more strongly associated with cochlear-weighted audiometric impairment. The consistency of findings across air-conduction thresholds, bone-conduction thresholds, and speech measures suggests that audiometric indicators of cochlear involvement are more closely related to disease chronicity than to cholesteatoma presence alone. These results highlight the importance of early disease control and timely management to limit potential cochlear-weighted deterioration, irrespective of cholesteatoma status.
